# Effect of Four Groups of GO-CF/EP Composites with Ideal Infiltration Structure and Different Layering Ways on Damping Properties

**DOI:** 10.3390/polym14122358

**Published:** 2022-06-10

**Authors:** Feichao Cai, Soo-Ho Jo, Yuqin Ma, Haiyin Guo, Yi Xu, Wei Xu, Fei Li

**Affiliations:** 1 School of Power and Energy, Northwestern Polytechnical University, Xi’an 710072, China; caifeichao@nwpu.edu.cn; 2 Department of Mechanical Engineering, Seoul National University, Seoul 08826, Korea; jsh1201@snu.ac.kr; 3 Key Laboratory of Road Construction Technology and Equipment of MOE, School of Construction Machinery, Chang’an University, No.126, Middle Section of Erhuan South Road, Xi’an 710064, China; 4 School of Mechano-Electronic Engineering, Xidian University, No.2, Taibai South Road, Xi’an 710071, China; haiyin_guo@163.com (H.G.); yixu6399267@163.com (Y.X.); hntb_xuwei@aliyun.com (W.X.); lifeili1123@163.com (F.L.)

**Keywords:** layer way, GO-CF/EP composites, force hammer method, damping ratio, resonance frequency

## Abstract

In this paper, four groups of graphene oxide and carbon fiber hybrid-reinforced resin matrix (GO-CF/EP) composites with different layering ways were prepared by a vacuum infiltration hot pressing system (VIHPS). The damping properties of the specimens with different layering ways were tested by the force hammer method, and the micromorphology of the specimens was photographed by scanning electron microscope. The experimental results showed that the damping properties of GO-CF/EP composites gradually increased with the increase in the number of Y-direction layers. The [XYXYXY]_6_ has the best damping property, with a damping ratio of 1.187%. The damping ratio is 5.3 times higher than that of [XXXXXX]_6_ layer mode, and the first-order natural frequency is 77.7 Hz. This is mainly because the stiffness of the X-direction layer is larger than that of the Y-direction layer, and its resistance to deformation is considerable. Therefore, its decay rate is slower. The Y-direction layer has weak resistance to deformation and fast energy attenuation. The increase in the number of Y-direction layers will lead to the overall increase in, and the improvement of, the damping properties of GO-CF/EP composites.

## 1. Introduction

Carbon fiber reinforced resin matrix (CFRP) composites have significant advantages of a lightweight, high-heat resistance, good corrosion resistance, and high strength, and are ideal high-performance materials in important technical fields such as aerospace, weapons, and equipment [1,2], especially in the leading-edge aerospace field, such as wing housing, helicopter, propeller blade, and engine duct design. Moreover, composites play a role in major structures, such as complete wing and fuselage components. Civil and military structural dynamic systems are prone to serious vibration when they are close to resonance, limit cycle, chaos, and aeroelastic instability conditions. Enhancing the damping of the structural system can not only reduce aeroelastic flutter and gust load but also improve the fatigue life, cockpit noise, maneuverability, and operational performance of structural components [3,4]. Therefore, the analysis and effective prediction of the damping performance of composite materials can realize the effective control of structural vibration and impact, noise, and fatigue failure, which has very important engineering practical significance [5]. For this reason, many experts and scholars have carried out in-depth and multifaceted academic research studies on the damping performance of CFRP and achieved certain research results.

Among them, Rahman et al. [6] prepared flax fiber reinforced polypropylene (FFRP) composites with different fiber orientations through the vacuum bagging process and tested damping performance by the force hammer method. It was found that with the increase in layering angle, the damping increased. It was concluded that the damping decreases slightly at 45°. Florence et al. [7] used vacuum bag-forming technology to prepare sandwich plates of 70% CF and 30% E-glass fiber. The influence of different fillers on the damping performance was explored. It was found that the polyurethane (PU) filled sandwich plate has good damping performance. Bhudolia et al. [8] prepared thermoplastic tubular composites using the balloon-assisted resin transfer molding (B-RTM) manufacturing process and conducted vibration modal analysis and test. The results showed that the structural damping of tubular composites exhibits different results at different output positions. Tsimouri et al. [9] fabricated sandwich CFRP structures with polydimethylsiloxane (PDMS) as the core layers. The high damping of the structures was achieved by exploiting the high shear deformations induced within thin PDMS layers. Although the composites were successfully prepared by the above processes, the microstructure of the composites was not characterized, and the influence of the preparation methods on the composites was not systematically explored. A vacuum infiltration hot pressing system (VIHPS) independently developed and designed by the team was adopted in this paper [10]. In this system, electromagnetic stirring, ultrasonic dispersion, vacuumization, hot pressing infiltration, and other methods were used to prepare graphene oxide and carbon fiber hybrid-reinforced resin matrix (GO-CF/EP) composites with ideal structure.

Graphene, a nanomaterial, is increasingly being used to improve the damping of CFRP because of its excellent mechanical properties and large specific surface area [11,12,13]. Sarikaya et al. [14] studied the vibration damping behavior of graphene nanocomposites through dynamic mechanical analysis and cyclic tensile compression test. The experimental results showed that the damping performance of the composite could be improved by about 70% by adding graphene nanoplatelets (GNP). Aside from the interfacial stick–slip friction between nanomaterials and matrix, interfacial sliding inside the nanomaterials has also attracted interest in improving the energy dissipation in composites. Lu et al. [15] fabricated sandwich PU beams with GNP as the core layers. The damping loss factor of these composites measured from quasistatic and dynamic damping tests increased by 71% and 94% compared with the analogous values from PU beams, respectively. They found that the wrinkles play an important role in energy dissipation caused by the inner interfacial slips of GNP. Moreover, they fabricated PU foam skeletons with multilayer sandwich GO coatings [16]. The stick–slip sliding of GO/PU and a combination of GO interfacial sliding and the PU intrinsic damping capacities had a synergistic effect on damping enhancement. Matsubara et al. [17] prepared a damping material using natural rubber (NR) as the matrix and micron-sized polyethylene terephthalate (PET) as the filler. The loss factor was determined by dynamic mechanical analysis, and a three-dimensional strain map was obtained using marker tracking in X-ray-computed tomography (CT) data. The results showed that adding 5% PET fiber into NR can improve the loss coefficient of NR, and the nonlinear damping of the composite rubber is affected by the separation of the filler–matrix interface and the internal strain of the material.

In addition, many experts and scholars have made achievements in the factors affecting the damping performance of composite materials. Chen et al. [18] analyzed the effects of different inclined ripple angles and fiber orientations on the natural frequency and loss factor of CFRP 3D double-arrow (3D DAH) heteromorphic metamaterials by using the energy method. The results showed that the natural frequency of CFRP is mainly determined by the stiffness vibration of 3D-DAH metamaterials along the main direction, and the loss coefficient is the largest around 45° fiber angle. Rueppel et al. [19] studied the layering angle of CF and flax fiber composite laminates and tested them through logarithmic decrement measurement, dynamic mechanical analysis, and vibration beam measurement. It was concluded that the damping performance is improved, and the elastic modulus decreases with the increase in layering angle. Assarar et al. [20] found that the damping properties of flax carbon mixtures with an external flax layer increased with the increase in the flax fiber content. Fairlie and Njuguna [21] also reported that adding two external flax fiber layers improved the damping performance of CFRP by 94%. Zhang et al. [22] studied the influence of volume fraction of E-glass fiber and layering angle on damping and strength of composite laminates. The experimental results showed that the damping capacity of composite laminates could be improved by increasing the fiber laying angle and decreasing the volume fraction of E-glass fiber, but the tensile strength of composite laminates would be decreased. Al-Obaidi et al. [23] studied the mechanical properties of leaf spring composites. The results showed that the bearing capacity of the composite leaf spring is related to the laying angle and volume content of the fiber. The matrix type has a significant effect on the stiffness. Nishant Varma et al. [24] showed that the natural frequency of composite leaf spring is 93% higher than that of leaf spring. The effects of different layering methods and chemical treatments on mechanical properties and free vibration properties of composites were discussed in the literature [25]. Table 1 is a comparative table of recent research results on the damping properties of composite materials. Most of the above studies are about the influence of fiber content and layering angle on the damping performance of composite materials. However, there are few studies on the effect of unidirectional carbon fiber layering on the damping properties of composites. Therefore, it is necessary to investigate the effect of layering on the damping properties of composite materials. The damping of structure can be affected only by changing the way of layering, which provides a new idea for the design and application of composite materials.

In this paper, four distinct groups of GO-CF/EP composites with ideal infiltration structures and different layering ways were prepared by VIHPS. The microstructures of the prepared samples were well impregnated without holes and stratification. The damping performance was investigated by force hammer test and microscopic morphology observation. This research is of great significance to aerospace [26], building materials or automotive [27], and machine tool [28] fields.

## 2. Materials and Methods

### 2.1. Test Materials

In the experiment, the reinforcement was a 2D-T700 CF cloth purchased from Toray Company (Tokyo, Japan). GO was purchased from Shenzhen Suiheng Technology Co., Ltd. (Shenzhen, China). The matrix is diglycidyl ether bisphenol A type E51 epoxy resin, the curing agent was phenolic amine T31 curing agent, and both were purchased from Nantong Star Materials Co., Ltd. (Nantong, China). Anhydrous ethanol was purchased from Chinasun Specialty Products Co., Ltd. (Changshu, China).

### 2.2. Preparation Process of Specimens

#### 2.2.1. Preparation of Curing Mixed Solution Containing GO

**Figure 1:**
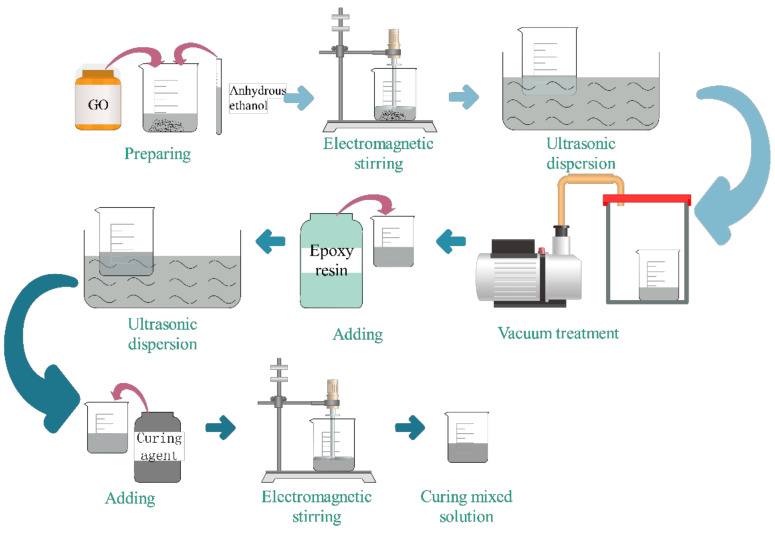
Preparation flow chart of curing mixed solution containing GO.

As shown in Figure 1, the specific preparation process of curing mixed solution containing GO was as follows: (1) Firstly, an appropriate amount of GO powder and anhydrous ethanol was weighed into a beaker and stirred for 5 min; (2) The solution was placed in an electromagnetic mixer and stirred electromagnetically at 500 rpm for 30 min. The aim was to make GO powder disperse more evenly in anhydrous ethanol; (3) The solution was dispersed in an ultrasonic cleaning machine with the ultrasonic frequency of 40 kHz, power of 200 W, and time of 40 min; (4) The dispersed GO solution was poured into the weighed epoxy resin and stirred with a glass rod; (5) Then, it was placed in an electromagnetic stirrer and stirred electromagnetically at 500 rpm and a time of 60 min. The purpose was to make GO evenly dispersed in the epoxy resin; (6) It needed to be placed in an ultrasonic dispersion cleaning machine with a frequency of 40 kHz, a power of 200 W, and a time of 60 min; (7) The solution was put into the vacuum oven for vacuumization, the vacuum degree was −0.09 MPa, and the time was 24 h. The purpose was to remove the bubbles generated in the solution during the preceding steps; (8) The proper amount of curing agent was added to the solution. The mixture was first stirred evenly with a glass rod and then placed in the electromagnetic mixer for electromagnetic stirring. The speed was 500 rpm, and the time was 15 min.

#### 2.2.2. Preparation of GO-CF/EP Composites

**Figure 2:**
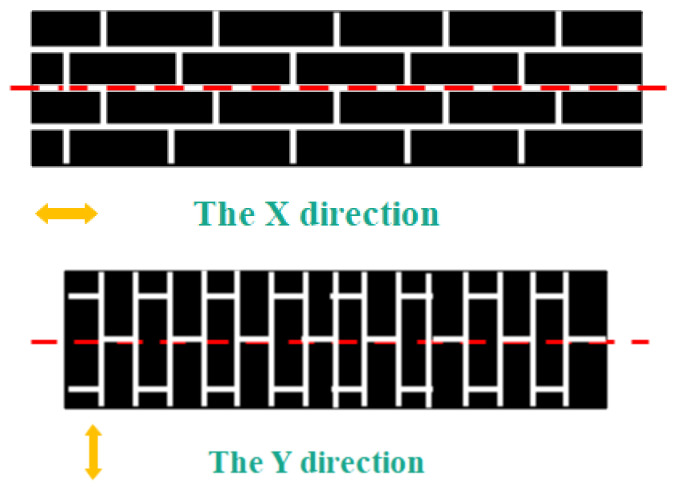
Schematic diagram of fiber orientation.

**Figure 3:**
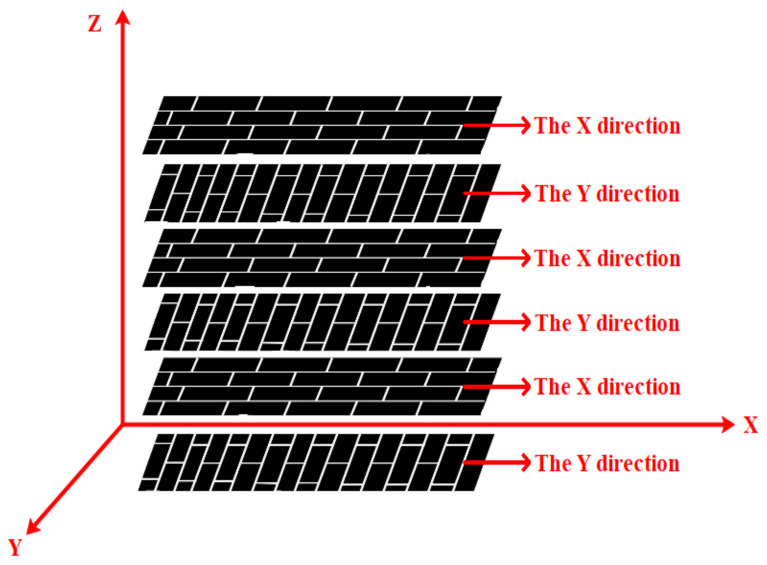
Schematic diagram of layering [XYXYXY]_6._

**Figure 4:**
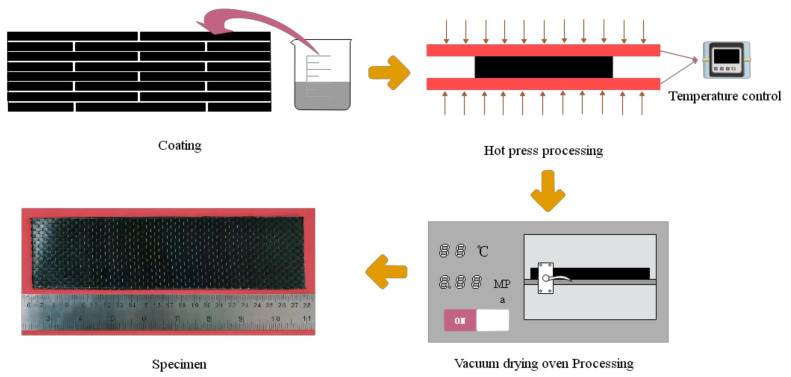
Preparation flow chart of GO-CF/EP composites.

After the preparation of curing mixed solution containing GO, we prepared the GO-CF/EP composite sample. The specific process was as follows: (1) A brush was used to evenly apply a curing mixture containing GO to both sides of the cut CF. The fiber orientation that is parallel to the axial direction was X-direction, and perpendicular to the axial direction was Y-direction, as shown in Figure 2; (2) In order to obtain CF preforms, the coating CF needed to be stacked in a layered way. Figure 3 is the layering diagram with the layering mode of [XYXYXY]_6_. In the stacking process, it was necessary to pay attention to the neat stacking of CF and the discharge of bubbles; otherwise, it would cause defects such as CF shift and stratification; (3) The CF preform was transferred to the molding press, and 0.7 MPa mold pressure was applied at 50 °C for 10 min; (4) The CF preform was moved from the molding machine to a thermostatic drying vacuum oven preheated to 90 °C, and heated and cured for 20 min under the vacuum condition of −0.09 MPa; (5) CF preforms are removed from a thermostatic drying oven and cooled at room temperature. The specimens of GO-CF/EP composites are obtained. The specific preparation process is shown in Figure 4.

### 2.3. Test and Characterization Methods

**Figure 5:**
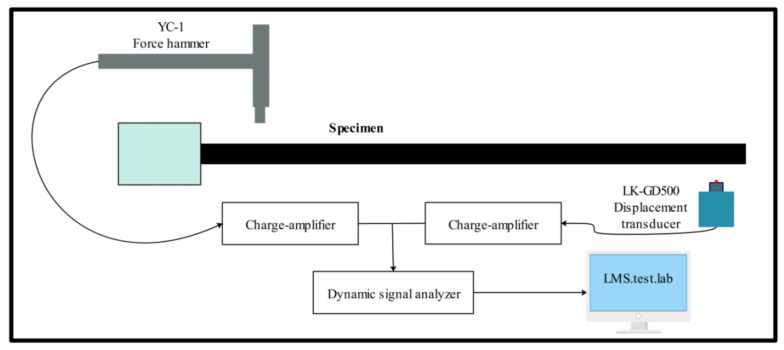
Schematic diagram of vibration model test system.

**Figure 6:**
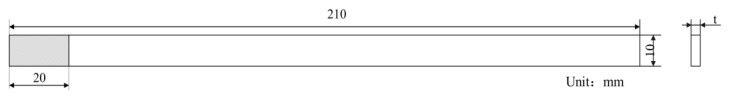
Schematic diagram of the size of the test piece.

According to GB/T 18258-2000 standard, a force hammer of SN L W37727 (PCB Piezotronics, Inc., Beijing, China) with a sensitivity of 2.267 mV/N was used at room temperature. The displacement sensor model was LK-GD 500 (Senmeirui Technology Co., Ltd., Shenzhen, China). The charge amplifier model was YE5873A (Jiangsu Lianneng Electronic Technology Co., Ltd., Yangzhou, China), and the signal collector was LMS SCADAS Mobile. Ms. Test. Lab (Beijing Ruide Hengxin Technology Co., Ltd., Beijing, China) was used for data analysis. The size of the sample was 210 mm × 10 mm × 2 mm. There were four specimens of GO-CF/EP composites with the same layer way. The schematic diagram of the vibration modal test system is shown in Figure 5 and Figure 6 is the schematic diagram of the specimen size of the vibration modal test.

The test process was as follows. One end of the sample is fixed onto the fixture. The clamping length was 20 mm, and the cantilever length was 190 mm. The position of the displacement sensor needed to be adjusted so that infrared light was projected near the sample axis. The force hammer gently hammered the sample on one side near the fixture, and the impact point was as far away from the axis of the sample as possible. The number of intermittent impacts was five, and the difference between the five impact forces was as small as possible. The change of displacement response with time was measured. When the specimen was impacted, the vibration amplitude of the specimen farthest from the fixture fluctuated, and the infrared ray sent by the displacement sensor captured the vibration signal and transmitted the signal to the data collector. The data collector recorded the readings of the displacement sensor on the computer and then calculated the frequency–response function using Fast Fourier Transform in special software LMS Test. Lab. In order to reduce the impact of noise signals during the test, the signal-to-noise ratio of the input pulse signal was improved by adding a force window, the influence of noise was reduced by adding an exponential window to the response signal, and the noise of transfer function was eliminated by averaging technique. The damping calculation formula is shown in Equation (1) [29].

(1) $$\ln\left(\frac{A_{n}}{A_{n+1}}\right)\approx 2\pi \zeta$$

where *A_n_* and *A_n+_*_1_ are the vibration amplitudes of the *n*th and the *n +* 1th; *ζ* is the damping ratio.

## 3. Analysis of Test Results

### 3.1. Microstructure

#### 3.1.1. Microstructure of GO

**Figure 7:**
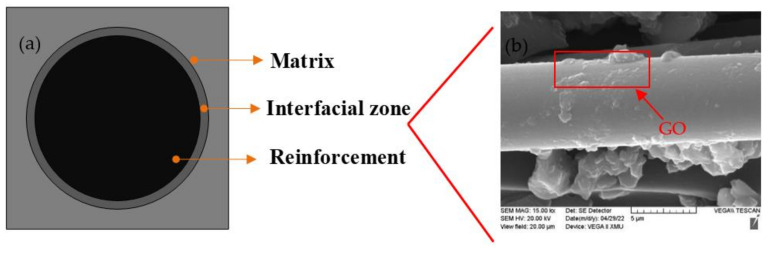
Schematic diagram of the three-phase model. (**a**) Three-phase model; (**b**) Mechanical locking structure.

The interface bonding zone is the bonding transition zone between the reinforced fiber and the resin matrix, as shown in Figure 7a, with a certain thickness. Different from the reinforced fiber, it has a relatively high shear modulus, which provides necessary conditions for energy dissipation. In CFRP composites, the energy consumption at the interface between fiber and matrix is very poor [30]. Therefore, the improvement of its performance will inevitably affect the damping performance of CFRP. In this paper, GO was added to improve the properties of the interface binding zone. It was found by SEM that GO was distributed on the fiber bundle in the form of folds, as shown in Figure 7b. Obviously, it can be observed that the fiber bundles no longer appear in a smooth form but have many rough GOs on their surface, forming a mechanical locking structure. This phenomenon will lead to increased internal friction and faster energy consumption in the process of energy dissipation. In the interface bonding zone, the movement of boundary phase molecules needs to overcome resistance, which takes a certain amount of time, resulting in boundary phase damping. The addition of GO will increase the damping of this bounded phase. In addition, the internal friction force will be generated by the debonding and slippage of the boundary phase under the action of external force, which will also affect the damping of the material, thus improving the damping performance.

The influence of filler on the damping property of polymer material mainly comes from two aspects: one is the influence of the damping property of filler, and the second is the influence of the shape of filler. In general, the specific surface area of the filler is different due to its different shapes and types, so its influence on damping performance is also different. GO sheets have a high specific surface area and relatively low particle density [31], which contributes greatly to the damping of polymer materials. The folded structure is located between the fiber and the resin, showing the damping of the micro-constraint layer. That is, the Van der Waals interaction between the GO sheets is much weaker than the bonding between CF and epoxy, so the interfacial sliding of the multilayer GO can be activated by the deformation of the fiber–matrix interface. The larger fiber–matrix interface area and multilayer sliding deformation in GO can generate more energy dissipation [32]. Furthermore, the slippage between graphene layers is recoverable under small deformations [19], thus enabling the material to dissipate energy continuously without structural deterioration. Because the orientation of GO in the epoxy resin matrix is random, there are many sliding phenomena at the interface between GO and resin. This improves the vibration energy loss capacity of the system. In addition, fillers limit the movement of polymer chains and increase the internal friction of the material. The relative lag between strain and stress increases, the internal friction of the material improves, and the effective damping temperature domain of the material expands. These phenomena can well explain the reason why the damping performance of GO-CF/EP composites is better than that of CFRP.

#### 3.1.2. Micromorphology of Infiltration Structure

**Figure 8:**
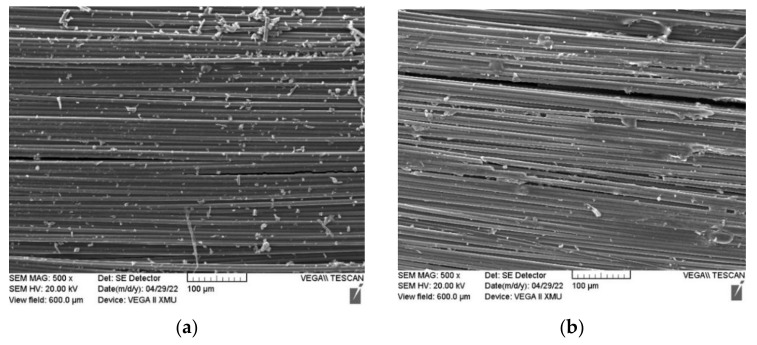

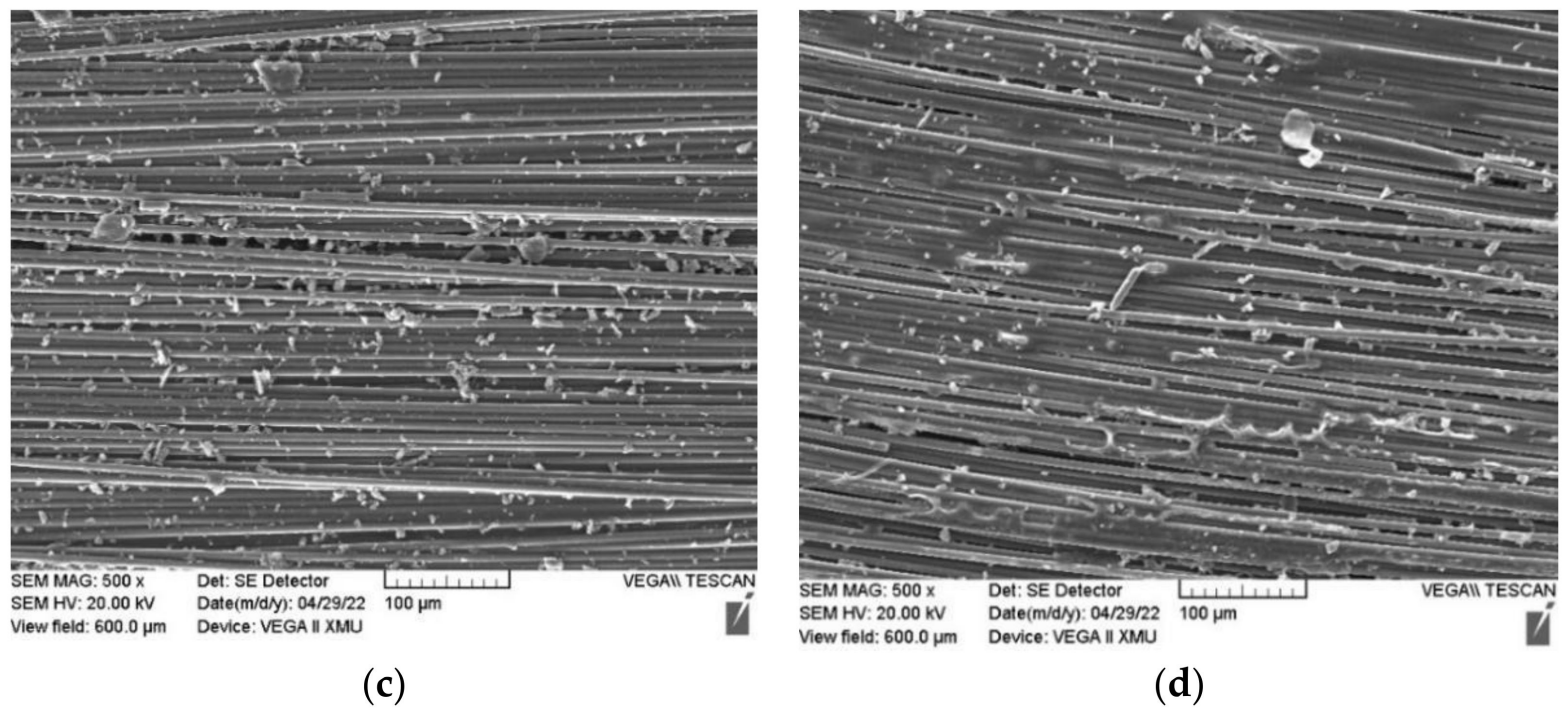
Morphology of microinfiltration of GO-CF/EP composites. (**a**) [XXXXXX]_6_; (**b**) [XYXXXX]_6_; (**c**) [XYYXXX]_6_; (**d**) [XYXYXY]_6_.

In order to explore the mechanism of the influence of layer way on the damping performance of the prepared GO-CF/EP composite, SEM images of four groups of samples with different layering ways were taken, as shown in Figure 8. It is obvious that the infiltration effect is ideal; the matrix is evenly distributed in the composite. The fiber is tightly combined with the matrix, showing bright black, and there are no obvious defects such as fat poor and fat rich, bubbles, holes, and so on. GO is evenly distributed on the fiber. When subjected to impact force, relative sliding occurs between GO and matrix as well as between GO and GO. The addition of GO can improve the toughness of the resin curing system and enhance the impact resistance of the composite system. The toughening mechanism may be due to the heterogeneity of the solidified cross-linking network, resulting in the formation of a micro heterogeneous continuous two-phase structure to achieve energy dissipation and thus significantly improve the impact resistance of composites. There are no obvious microstructure defects in the morphology of different layering ways, so the damping performance is mainly affected by different layering ways.

### 3.2. Damping Performance

#### 3.2.1. The Damping Ratio

**Figure 9:**
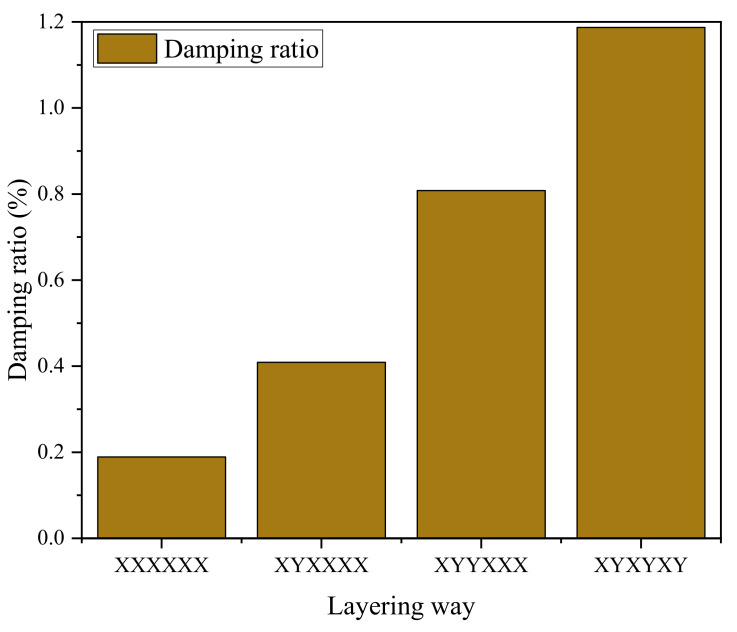
Damping ratios of different layering ways.

**Figure 10:**
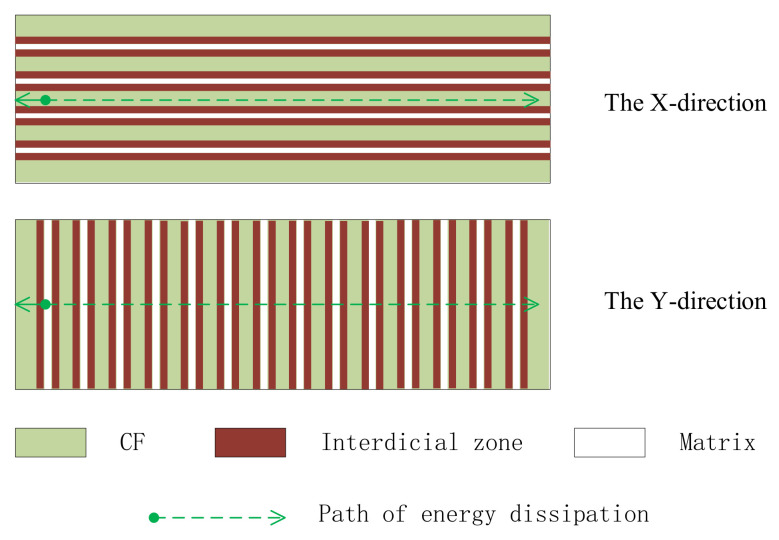
Diagram of energy dissipation.

Damping reflects a material’s ability to convert mechanical energy into heat energy. The greater the damping, the greater the rate of energy loss through the material. At the same time, a greater damping material can significantly reduce the amplitude peak of the system in resonance. According to the test results of the force hammer method, the free attenuation curves of different layering ways are obtained. Obviously, it can be observed that the attenuation of vibration amplitude is faster and faster with the increase in the Y-direction layer. Its damping ratio is plotted as a bar graph, as shown in Figure 9. [XXXXXX]_6_ has the lowest damping ratio of 0.189%. The damping ratio of [XYXYXY]_6_ is 1.187%, which is 5.3 times higher than that of the specimen without the Y-direction layer. This data conclusion is also consistent with the view in reference [19,33]. This is mainly because when the specimen is subjected to impact force, the energy dissipation path of the specimen is fixed, as shown by the arrow in Figure 10. However, the damping properties of GO-CF/EP composites with different layering ways are different. In the X-direction fiber layer, the energy dissipation path is consistent with the direction of the fiber. Therefore, its damping property is mainly affected by the damping property of the fiber. Although the reinforced CF can play a role in damping when subjected to impact force, the material property is rigid, and, coupled with its own viscoelastic difference and poor interface damping, its damping coefficient is low. Therefore, the contribution of its inherent damping to the damping of the specimen is not obvious, so the specimen with [XXXXXX]_6_ layering mode has the smallest damping ratio. Similarly, in the Y-direction layer, the dissipation path of energy is consistent and fixed. However, the damping material in its path changes. In the Y-direction layer, the part of matrix participation has increased. The contribution of the matrix to the overall properties of composites plays an important role [22,23]. It can be said that the fiber is combined with the matrix, so the damping performance of the matrix will have a great influence on the damping performance of the specimen. In addition, in CFRP, the organic resin is a typical viscoelastic material with a large damping coefficient, and its polymer characteristics tend to produce energy consumption. Its contribution to the damping ratio is much higher than that of other components. As a result, the increase in resin matrix participation rate has a great change in the damping performance of specimens. Therefore, with the increase in the number of Y-direction layers in GO-CF/EP composites, the damping ratio presents an almost linear increase, and the specimen with [XYXYXY]_6_ layers has the highest damping ratio.

#### 3.2.2. Natural Frequency

**Figure 11:**
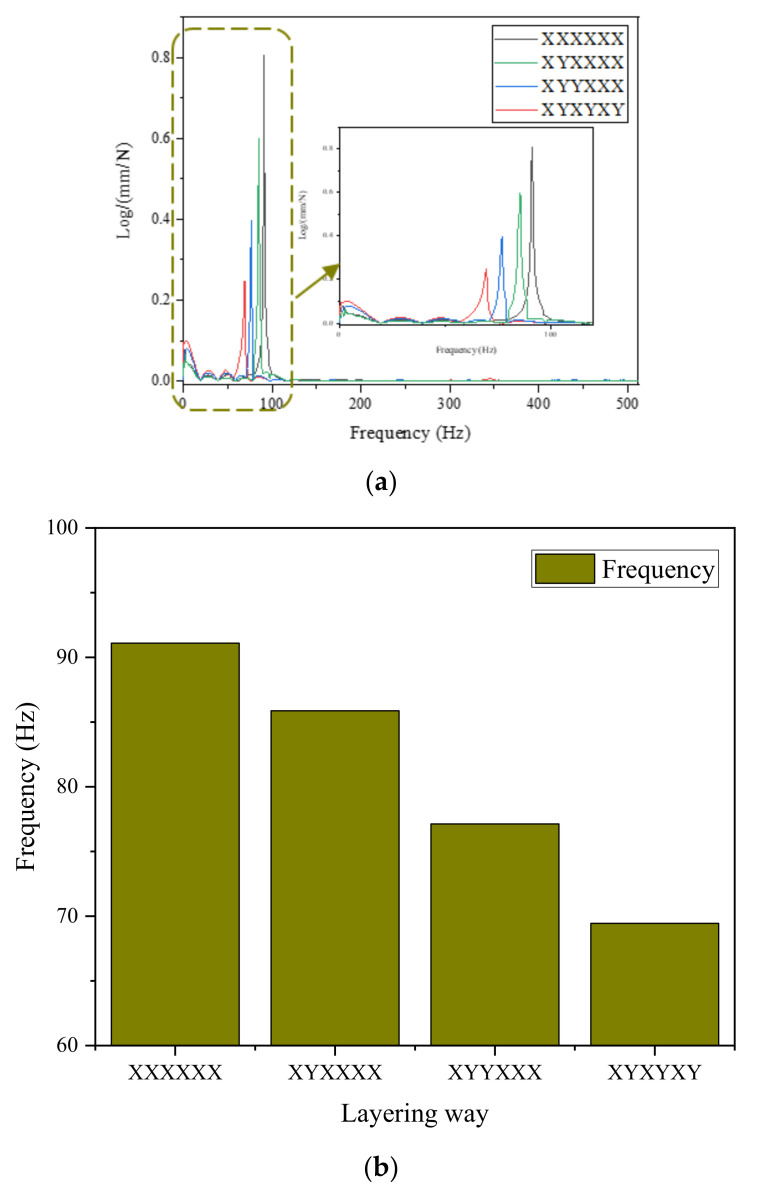
The first-order resonance frequency of different layering ways. (**a**) Frequency–response curve; (**b**) The first-order resonance frequencies of different layering ways.

The first-order resonance frequency of laminates is a key index to measure the vibration characteristics of structures. The higher the fundamental frequency, the higher the overall stiffness, the better the stability of the laminates, and the stronger the deformation resistance under the action of pulse load, which can reduce the incidence of resonance of the laminates. The first mode is determined by the inherent damping properties of the material, such as viscoelasticity. The first-order resonance frequency of GO-CF/EP composites prepared was tested by the force hammer method. In the range of 0–2000 Hz, only the first-order resonance frequency is obvious. Therefore, in the next step, the acquisition frequency is uniformly set as 0–512 Hz. The first-order resonance frequency–response curves of specimens with different layering ways were obtained, and the results were plotted to obtain the curves shown in Figure 11a. It is observed that with the increase in the number of Y-direction layers, the corresponding frequency of peak values in the response curve of the specimen gradually moves to the left, and the corresponding peak values gradually decrease. The values of first-order resonance frequency under different layering ways are drawn as a bar chart, as shown in Figure 11b. It is observed that the first-order resonance frequency of the specimen decreases gradually with the increase in the number of Y-direction layers in the specimen. The reason for this phenomenon is that with the increase in the number of Y-direction layers, the modulus of the composite decreases after forming. At the same excitation, the material amplitude increases, and the natural frequency decreases. The natural frequency of CFRP mainly depends on the stiffness vibration in the main direction of the material, while the flexural stiffness of the laminates decreases with the increase in the number of fiber laminates in the Y-direction, resulting in a downward trend of the natural frequency. The structure stiffness of Y-directional fiber is small, and energy is consumed mainly by internal friction between fiber and matrix. Small amplitude in the vibration spectrum indicates high damping [34]. By observing the frequency–response curve, it is found that with the increase in the number of Y-direction layers, the rising trend of the response curve at the peak gradually slows down. The response curve amplitude of layering mode [XYXYXY]_6_ is significantly lower than that of layering mode [XXXXXX]_6_, and the slope of its peak curve becomes slow, so its damping performance is the best. This also confirms that the steeper the frequency–response curve, the smaller the loss coefficient, and the weaker the damping capacity. The smoother the frequency–response curve, the greater the damping coefficient and the better the damping performance of the corresponding material. In addition, increasing the number of Y-direction fibers will increase the structural flexibility, which will also lead to a reduction in natural frequency. These conclusions are also confirmed in reference [18,23].

## 4. Discussion

The damping and vibration reduction characteristics of GO-CF/EP composites prepared in this paper mainly come from the intrinsic damping of resin, CF, and additive GO, the boundary phase damping of resin-GO and resin-fiber, viscoelastic damping, thermoelastic damping, etc. The volume fraction of fiber is generally higher, and the fiber widely existing in the resin matrix can increase the strain of the composite and improve the energy consumption capacity of the material. The main dissipation mechanism of GO-CF/EP composites is friction sliding. Compared with traditional reinforcement materials, the friction area between GO and resin increases with the addition of GO, so its dissipation mechanism is higher.

In the multiphase coexistence system of composite materials, the interface refers to the contact area between the reinforcement phase and the matrix phase. The interface layer is different from the adjacent reinforcement phase and the matrix phase in structure and properties, and the properties change in the direction of thickness to a certain extent. In composite materials, the interface is the core structure. It is widespread and abundant and divides the large continuous body into several small continuous regions, causing discontinuity of structure and performance at the interface. Different from the damping mechanism of general materials, the energy dissipation of fiber-reinforced composites is caused by different mechanisms at the structural level, such as viscoelasticity between the matrix and the fiber material, damping at the interface of the fiber and matrix, etc. In terms of laminates, the damping of composite materials strongly depends on the characteristics, orientation, interlayer effect, and vibration coupling of the constituent layers [35]. With the increase in the number of Y-direction layers, the interlayer effect and vibration coupling will change. This reason has been explored.

Yim et al. [36] found that increasing the percentage of X-direction layers in the laminates can reduce damping, even though the viscoelastic effect of the matrix is the main damping mechanism. Adams et al. [37] found that for cross bedding, the most important factor is to determine the cross-bedding ratio of the relative number of X-direction and Y-direction layers. These also confirm the importance of exploring the damping performance of different layering ways. The energy dissipation of composite laminates mainly depends on the inconsistencies of compressive deformation between upper and lower panels when the structure is deformed under stress. The interlaminar shear stress between layers can dissipate the vibration energy of the structure. For composite laminates, the bending deformation perpendicular to the fiber direction and the shearing deformation within the laminate are the main reasons that affect the damping characteristics. Transverse damping factor and shear damping factor are the most important factors affecting the damping ratio of composite laminates. The stress-related damping in the fiber direction is almost entirely determined by the fiber damping. The damping related to the stress perpendicular to the fiber axis and the damping related to the in-plane shear stress are almost entirely determined by the matrix damping. The fiber moves in a viscoelastic matrix in longitudinal and transverse dissipation. The comparison of experimental results shows that the fiber–matrix interaction is dominant in the longitudinal direction [38]. All these indicate that the damping performance of the Y-direction layer is stronger than that of the X-direction layer. Therefore, with the increase in the number of Y-direction layers, the damping performance of GO-CF/EP composites will be gradually enhanced, and the damping ratio will also be significantly improved.

## 5. Conclusions

In this paper, VIHPS was used to prepare four groups of GO-CF/EP composites with ideal infiltration structures and different layering ways. Their damping ratio and first-order resonance frequency were collected. Through the combination of SEM results and experimental data, the following results were obtained:

(1) The addition of nanoscale GO can significantly enhance the damping performance of CFRP composites. The main dissipation mechanism of GO-CF/EP composites is friction sliding. Compared with traditional reinforced materials, the addition of GO makes the friction area between GO and resin larger, so the dissipation mechanism is higher;
(2) Among the prepared specimens, the damping ratio of [XYXYXY]_6_ specimens is up to 1.189%, which is more than six times that of [XXXXXX]_6_ specimens. This is because, with the increase in the number of Y-direction layers, the participation of the matrix, which contributes greatly to the damping performance, gradually increases, thus improving the damping performance of GO-CF/EP composites;
(3) The natural frequency of GO-CF/EP composites is greatly affected by the stiffness of the materials. The anisotropy of the stiffness of the specimen changes with different lamination modes, and so does the dynamic stiffness of the laminate. With the increase in the number of Y-direction layers, the dynamic stiffness decreases, and the natural frequency of the material decreases.

## Figures and Tables

**Table 1:** A comparative table of recent research results on damping properties of composite materials.

Author	Craft	Materials	Conclusion	Reference
Rahman	Vacuum bagging process	Flax fiber reinforced polypropylene composites	The loss factor increases with increasing frequency and decreases slightly with increasing fiber content.	[6]
Florence	Vacuum bag molding technique	Sandwich plates with a hybrid fiber of 70% CF and 30% of ‘E’ glass fiber	The polyurethane-filled sandwich plate had a higher damping performance than the Rohacell-filled panel and wheat husk-filled panel.	[7]
Bhudolia	B-RTM	Carbon–Elium composites	At different output positions in the tube, the structural damping of the carbon–Elium composite was improved by 21.7%.	[8]
Sarikaya	Solution mixing process	“single-layer graphene” and “GNP”	The addition of GNP will increase the damping properties of the nanocomposites by up to ~70%.	[14]
Wenjiang Lu	Modified dip-coating process	Open-cell PU foams containing multilayered GO	These engineered composite foams with extremely low GO content (−0.12 wt%) afford a significant increase in quasistatic energy dissipation (52%) and dynamic damping (76%) when compared with counterpart foams coated with the same number of pure PU dispersion layers.	[16]
Yun-Long Chen	Hot-press compression molding method and layer-by-layer assembly method	CFRP 3D DAH auxetic metamaterials	The natural frequency of CFRP 3D DAH attached metamaterials is mainly determined by the stiffness of the main vibration direction, and the loss factor is the highest when the angle of the layer is about 45°.	[18]
Rueppel	Autoclave manufacturing and compression resin transfer molding	CFRP and FFRP	The damping of the two materials increases with the increase in the angle. The matrix and interface seem to be primarily responsible for damping at lower frequencies.	[19]
Assarar	Platen press process	Flax–carbon twill epoxy composites	[C/F/C/C/F/C] laminates are 15% higher than non-hybrid carbon laminates without losing specific bending modulus.	[20]
Bao Zhang	Vacuum infusion molding process	Glass fiber reinforced composites	The 0° fiber layer is beneficial for improving the strength of the laminate. The 90° fiber layer is beneficial for the damping performance of the laminate.	[22]

## Data Availability

The data that support the findings of this study are available from the corresponding author upon reasonable request.
